# Long-term outcomes and quality of life after sleeve gastrectomy with major complications

**DOI:** 10.1007/s13304-026-02633-7

**Published:** 2026-04-04

**Authors:** Matan Shimron, Adam Abu-Abeid, Rotem Kariv, Anat Bendayan, Andrei Keidar, Sigal Fishman, Mati Shnell, Shai Meron Eldar

**Affiliations:** 1 Division of General Surgery, Tel Aviv Medical Center, 6, Weizman St., Tel Aviv, Israel; 2 The Gray’s Faculty of Medical Health and Sciences, Tel Aviv University, Tel Aviv, Israel; 3 Koret School of Veterinary Medicine, Hebrew University of Jerusalem, Jerusalem, Israel; 4 Department of Gastroenterology, Tel Aviv Sourasky University Medical Center, Tel Aviv-Yafo, Israel

**Keywords:** Bariatric surgery, Postoperative complications, Surgical outcomes, Weight loss, Quality of life

## Abstract

Sleeve Gastrectomy (SG) is the most common bariatric procedure worldwide. Its relative high safety profile is one of the reasons for its popularity. Despite this, SG may lead to short and long-term morbidity, sometimes severe, which may negatively impact patients’ quality of life (QoL). Data on this long-term impact of surgical complications is limited. Our objective is to determine the long-term outcomes and overall QoL in patients who had major postoperative complications after SG. This is a retrospective analysis of patients who underwent SG between the years 2010–2013 at single-tertiary center and completed a 10-year follow-up period. Data of patients with major complications was captured and matched to patients with an uneventful postoperative course based on a propensity score. Data withdrawn included weight trends, QoL measures, and obesity associated medical problems. Thirty patients with major complications after SG were matched to a control group of 45 patients with an uneventful postoperative course. There was no difference in baseline patient characteristics. Long-term weight outcomes were similar—with mean excess weight loss (EWL%) of 63.33% in the study group versus 59.93% in the control group (*p* = 0.55) and mean total weight loss (TWL%) of 27.4 ± 7.66%, in the study groups versus 25.28 ± 14.14% in the control group (*p* = 0.49). Interestingly, QoL measures showed significant impairments in the study group, with a BAROS QoL score of − 0.06 compared to 0.72 in the control group (*p* = 0.001). Despite the presence of major postoperative complications after SG, long-term weight loss outcomes were not significantly different between the two groups; however, they are associated with a long-term decline in QoL. Further studies are needed to explore and clarify this data.

**Supplementary Information:**

The online version contains supplementary material available at 10.1007/s13304-026-02633-7.

## Key points

- The most common major complications after SG were leaks and abscess formation.
- Patients with a complicated course after SG have good metabolic outcomes.
- There was no difference in weight loss outcomes.
- The BAROS QoL questionnaire was significantly higher in noncomplicated patients.

**Supplementary Information:**

The online version contains supplementary material available at 10.1007/s13304-026-02633-7.

## Introduction

The worldwide prevalence of obesity has tripled since 1980, with nearly a third of the population now classified as overweight or obese, largely due to energy-dense diets and decreased physical activity. Obesity adversely affects nearly all physiological functions, increasing the risk of multiple disease conditions and negatively impacting quality of life and work productivity, with healthcare costs estimated at $2 trillion annually [1].

Sleeve Gastrectomy (SG) is nowadays the most commonly performed metabolic and bariatric procedure worldwide [2]. It has been shown in many reports that SG is associated with a high safety profile, sustained weight loss, and resolution of obesity associated medical problems [3–5]. Despite its positive impacts, SG may be associated with major postoperative complications.

Major complications following surgery can negatively affect patients’ quality of life. These patients may undergo several interventional procedures including reoperations and endoscopies, may have long hospitalization periods, and higher rates of postoperative mortality [6]. Following the early postoperative course patients may still require hospital readmissions and different treatment methods.

These repeated hospital visits, in addition to the financial and emotional strain may cause a significant negative impact on patients’ quality of life.

They may also experience different levels of dysphagia, nausea, vomiting and non-specific abdominal pain. Some develop cachexia and general fatigue.

The long-term outcomes for patients with a complicated course after SG are unclear as the literature is scarce.

The aim of this study is to assess long-term SG outcomes among patients with a major postoperative complication. These patients will be compared to a control group of patients with an uneventful postoperative course at least 10 years after surgery. The primary endpoint is to assess and compare the weight loss outcomes between groups. Secondary endpoints include resolution of obesity associated medical problems and quality of life parameters among both groups.

## Methods

This is a retrospective data analysis of patients with and without major postoperative complications after SG between 2010 and 2013 at a single tertiary center and completed a 10-year follow-up period. All patients with major postoperative complications and complete preoperative data were included. Patients were matched to a control group with a normal postoperative course based on body mass index (BMI) in a 1:1.5 ratio.

Major postoperative complications were defined according to the Clavien–Dindo (CD) classification as requiring surgical, endoscopic, or radiological intervention (CD3) or life-threatening complications requiring intensive care management (CD4) [7].

Preoperative data collected included—age at the time of surgery, gender, presence of type 2 diabetes, BMI prior to surgery, and prior abdominal surgeries including Laparoscopic Adjustable Gastric Banding (LAGB).

Perioperative data withdrawn included- intra operative complications, organ dysfunction, staple line leak, abscess/fluid collection formation, bleeding requiring blood products, endoscopic interventions, image guided drainage, reoperations, and ICU admissions. Staple line leak was considered when demonstrated by radiologic imaging (contrast extravasation), by drain content analysis (e.g., enteric content), or confirmed intraoperatively. Collections without radiologic or intraoperative proof of active leakage were categorized as abscess/fluid collection.

Postoperative data withdrawn included weight loss trends, metabolic outcomes, and quality of life measures according to the BAROS score.

### Quality of life measures

Participants were also asked to complete a quality-of-life questionnaire. Quality of life assessment was conducted using a questionnaire developed by Moorehead-Ardelt of the Bariatric Analysis and Reporting Outcome System (BAROS) protocol which is shown in supplementary Fig. 1 [8]. It consists of five domains: self-esteem, physical activities, social relationships, sexual activity, appetite, and work performance. For each domain there is one question, each with five response alternatives, representing a gradual level of satisfaction, with each answer varying from a minimum of − 0.5 to a maximum of 0.5. The questionnaire was administered over the phone and took approximately 10 min to complete.

### Statistical analysis

To minimize selection bias, propensity score–matched analysis was performed. Every patient from the complicated group was matched using their propensity score with the nearest neighbor patient with a non-complicated surgical course. The study group was matched in a 1:1.5 ratio to a control group according to patient BMI.

Data analysis was performed using the SPSS 15 software (SPSS, Inc.). Comparison between the two groups was made using an unpaired Student’s t-test. Categorical data was compared using a chi-square analysis with Yates correction and Fisher’s exact test, where appropriate. *P* < 0.05 was considered statistically significant. Data collected from the questionnaires was analyzed using statistical software. Descriptive statistics were used to summarize the data. Inferential statistics, such as regression analysis, was used to investigate the relationship between severe postoperative complications and quality of life outcomes.

## Results

During the study period, a total of 54 patients experienced major postoperative complications after SG. Of these, 3 patients were excluded because they subsequently underwent conversion to Roux-en-Y gastric bypass, and 1 patient died due to an unrelated malignancy. An additional 20 patients were either lost to follow-up or declined participation despite attempts at contact. Therefore, 30/54 patients (56%) were ultimately available and included in the long-term follow-up analysis (complicated SG group/ study group). The matched group (uncomplicated SG group/ control group) consisted of 45 patients. The mean follow-up time was 10–12 years.

### Baseline parameters

Baseline demographic and clinical characteristics were comparable between the two groups (Table 1). There were no statistically significant differences in age at the time of surgery (43.63 ± 9.71 vs. 39.97 ± 11.7 years, *p* = 0.16), sex distribution (20% male vs. 38% male, *p* = 0.09), prevalence of type 2 diabetes (16% vs. 20%, *p* = 0.68), or preoperative BMI (45.97 ± 7.31 vs. 45.01 ± 7.02 kg/m^2^, *p* = 0.57). Prior laparoscopic adjustable gastric banding (LAGB) and other non-bariatric abdominal surgeries were similar between groups, although a higher rate of prior non-LAGB abdominal surgery was noted in the uncomplicated group (18% vs. 3.3%, *p* = 0.056), approaching statistical significance.

**Table 1:** Baseline characteristics of uncomplicated SG compared to complicated SG

Parameter	Uncomplicated SG (n = 45)	Complicated SG(n = 30)	*P* value
Age, mean ± SD, year	39.97 ± 11.7	43.63 ± 9.71	0.16
Male, n (%)	17 (38%)	6 (20%)	0.09
T2D, n (%)	9 (20%)	5 (16%)	0.68
BMI before operation, mean ± SD, kg/m	45.01 ± 7.02	45.97 ± 7.31	0.57
s/p LAGB (%)	3 (6.8%)	3 (10%)	0.628
s/p other abdominal surgery	8 (18%)	1 (3.3%)	0.056

*SG* sleeve gastrectomy, *SD* standard deviation, *n* number, *BMI* body mass index, *T2D* Type 2 Diabetes

### Postoperative complications

Among the 30 patients in the complicated SG group, the most common complications included abscess/fluid collection (43.33%), staple line leaks (40%), reoperations classified as CD3b (53.33%) (Interventions under general anesthesia), and bleeding requiring packed cell transfusion (30%) (Table 2). Additional interventions included endoscopic procedures (26.66%), image-guided drainage (43.33%), and ICU admissions (6.66%). There were 18 reoperations in total (53.33% of the complicated group). Of these, 9 were performed due to postoperative bleeding and 9 due to staple line leaks.

**Table 2:** Major postoperative complications following SG

Complication	Total (n = 30)
Intraoperative complication, n (%)	1(3.33%)
Organ dysfunction, n (%)	3 (10%)
Staple line leak, n (%)	12 (40%)
Abscess/ fluid collection, n (%)	13 (43.33%)
Bleeding required PC, n (%)	9 (30%)
Endoscopic intervention (CD IIIa), n (%)	8 (26.66%)
Imaging guided drainage (CD IIIa), n (%)	13 (43.33%)
Reoperation (CD IIIb), n (%)	16 (53.33%)
Admission to ICU (CD IV), n (%)	2 (6.66%)

*SG* sleeve gastrectomy, *CD* Clavien Dindo, *PC* packed cells, *ICU* Intensive care unit

### Weight loss and metabolic outcomes

Long-term weight loss outcomes were not significantly different between the two groups, despite the presence of major postoperative complications. The mean total weight loss was 27.4 ± 7.66%, in the study groups versus 25.28 ± 14.14% in the control group (p = 0.49). The complicated group achieved a mean % excess weight loss (EWL) of 63.33 ± 15.27%, compared to 59.93 ± 28.92% in the uncomplicated group (*p* = 0.55). EWL points on the BAROS system were also similar (2.06 ± 0.58 vs. 1.84 ± 1.05, *p* = 0.29). Difference in improvement of medical conditions on the BAROS system was insignificant, as well (0.50 ± 0.97 vs. 0.52 ± 0.76, *p* = 0.91).

### Quality of life outcomes

Significant differences in quality of life (QoL) were observed between the two groups, favoring the uncomplicated group (Table 3). The BAROS QoL summary score was markedly lower in the complicated group (−0.06 ± 1.05) compared to the uncomplicated group (0.72 ± 0.92), with a statistically significant difference (*p* = 0.001). Subdomain analysis revealed lower scores in multiple QoL parameters among patients with complications, including physical activity (−0.15 ± 0.25 vs. 0.007 ± 0.26, *p* = 0.01), work performance (0.075 ± 0.26 vs. 0.2 ± 0.18, *p* = 0.01), and general well-being (0.001 ± 0.24 vs. 0.14 ± 0.26, *p* = 0.02). Other domains such as sex life (*p* = 0.05) and relationship with food (*p* = 0.06) showed lower score trends among the complicated group, although they did not reach statistical significance.

**Table 3:** Long-term QOL outcomes (BAROS score) after SG

	Uncomplicated SG (n = 45)	Complicated SG(n = 30)	*P* value
General well-being, mean ± SD	0.14 ± 0.26	0.001 ± 0.24	0.02
Physical activity, mean ± SD	0.007 ± 0.26	− 0.15 ± 0.25	0.01
Social, mean ± SD	0.17 ± 0.17	0.14 ± 0.22	0.49
Work, mean ± SD	0.2 ± 0.18	0.075 ± 0.26	0.01
Sex, mean ± SD	0.09 ± 0.2	0 ± 0.2	0.05
Approach food, mean ± SD	0.05 ± 0.47	− 0.1 ± 1.05	0.06
QOL summary points mean ± SD	0.72 ± 0.92	− 0.06 ± 1.05	0.001
EWL % mean ± SD	59.93 ± 28.92	63.33 ± 15.27	0.55
EWL points, mean ± SD	1.84 ± 1.05	2.06 ± 0.58	0.29
Medical condition points, mean ± SD	0.52 ± 0.76	0.5 ± 0.97	0.91

*SG* sleeve gastrectomy, *SD* standard deviation, *QOL* quality of life, *EWL* excess weight loss

## Discussion

The primary goal of bariatric surgery is to achieve substantial and sustained weight loss while reducing the burden of obesity-related comorbidities such as diabetes and hypertension. A loss of at least 50% of excess weight is commonly considered a successful outcome. In our study, both groups achieved considerable excess weight loss. As bariatric surgery continues to play a central role in the treatment of severe obesity—even in the evolving landscape that now includes pharmacologic therapies and endoscopic interventions—understanding its long-term outcomes and potential complications remains critically important [9]. Complications in bariatric surgery represent a major concern for both patients and surgeons, as they may lead to prolonged hospitalization, additional interventions, and increased healthcare utilization. The incidence of severe postoperative complications in this field is reported to be between 1.5 and 4.0% [10, 11]. Reported mortality after bariatric procedures usually does not exceed 0.1–0.2%. Although major postoperative complications after bariatric surgery are rare, they can result in prolonged hospital stays, higher readmission rates, increased healthcare costs, and mortality. Several studies have suggested that early postoperative complications—particularly severe ones requiring re-intervention or prolonged hospitalization—do not impair weight loss outcomes or diabetes and hypertension remission one year after bariatric surgery [12]. However, the longer-term impact has been less well-defined.

The findings of this study offer important insights into the long-term outcomes of Sleeve Gastrectomy (SG) among patients who experience major postoperative complications. Although the initial hypothesis posited that complications might adversely affect weight loss outcomes, the results demonstrate that long-term weight loss percentages were comparable between the complicated and uncomplicated groups. These results correspond with prior evidence showing that SG typically achieves durable weight reduction, even in heterogeneous patient populations [13, 14].

### Weight loss outcomes

The similar weight loss outcomes observed between the two groups reinforce previous findings that SG generates substantial and sustained weight loss over time [15, 16]. The absence of a significant difference in excess weight loss (63.33% vs. 59.93%, *p* = 0.55) aligns with reports suggesting that mechanical or anatomical complications do not necessarily diminish volume restriction and metabolic efficacy.

One possible explanation is that adherence to postoperative behavioral guidelines may remain intact even among individuals who experience complications. Past research has demonstrated that diet, physical activity, and psychological resilience play key roles in long-term weight outcomes [17, 18]. Future prospective studies should further examine these psychosocial and behavioral contributors, particularly in the context of postoperative morbidity.

### Quality of life implications

In contrast to weight loss, significant disparities were noted in QoL outcomes. The substantially lower BAROS score in the complicated group (− 0.06 vs. 0.72, *p* = 0.001) highlights the profound negative impact of complications on well-being. This is consistent with earlier work demonstrating the sensitivity of BAROS and other QoL tools to postoperative morbidity [8, 19].

Complications after SG—whether mechanical, infectious, or functional—can impose prolonged recovery demands, repeated hospitalizations, and psychological stress, all of which are known contributors to reduced QoL. Subdomain deficits in physical activity, work performance, and general well-being align with recent studies emphasizing that postoperative quality of life is closely linked not only to surgical success but also to emotional and social recovery [20, 21].

### Clinical implications and recommendations

These results underscore the necessity for enhanced preoperative counseling and strengthened postoperative surveillance. Previous research has emphasized the importance of realistic preoperative expectations and comprehensive patient education in improving satisfaction and outcomes [22, 23].

Given the marked QoL deterioration associated with complications, multidisciplinary postoperative support—including psychological care, nutrition counseling, and structured follow-up—should be considered standard practice. Studies have shown that structured support programs can significantly improve long-term QoL and reduce dropout rates [17, 21].

### Limitations and future research

The main limitations of this study include its retrospective design and relatively small number of patients experiencing complications, which may limit generalizability. Larger prospective cohorts are needed to more precisely assess the relationship between complications, weight loss, and quality of life.

Future research should also explore qualitative dimensions of the postoperative experience. Patient narrative studies have proven valuable in identifying unmet needs and faculty in postoperative support structures [21].

## Conclusion

In conclusion, while Sleeve Gastrectomy remains an effective long-term weight-loss intervention, major postoperative complications have a significant adverse impact on quality of life. These findings mirror earlier observations that QoL, rather than weight loss, may be the domain most sensitive to postoperative morbidity. A holistic approach that integrates metabolic outcomes with QoL support is therefore essential to optimizing patient care in bariatric surgery.

## Supplementary Information

Below is the link to the electronic supplementary material.

Supplementary file1 (JPEG 633 kb)

## Data Availability

Data supporting the fi ndings of this study are available from the corresponding author upon reasonable request.
