# Supplementary figures and images for: Long-term outcomes and quality of life after sleeve gastrectomy with major complications
Source: Updates Surg. 2026 Apr 4;78(4):1775–80. doi: 10.1007/s13304-026-02633-7 (PMC13421198; doi:10.1007/s13304-026-02633-7)

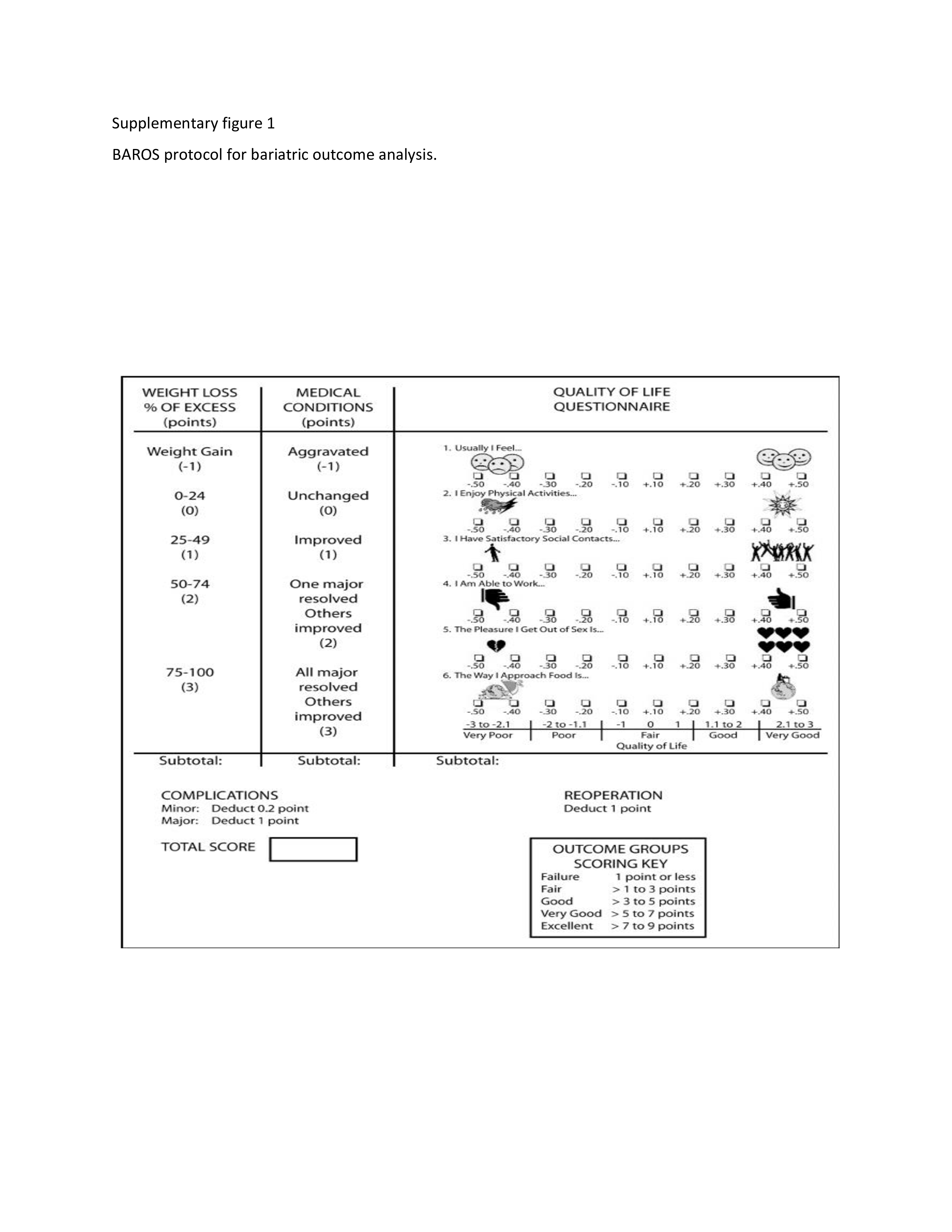

Supplement: Supplementary file 1 — Supplementary file1 (JPEG 633 kb) [file 13304_2026_2633_MOESM1_ESM.jpeg]
